# Vaccine induced memory CD8^+^ T cells efficiently prevent viral transmission from the respiratory tract

**DOI:** 10.3389/fimmu.2023.1322536

**Published:** 2023-12-18

**Authors:** Jinglin Zhou, Ida Uddback, Jacob E. Kohlmeier, Jan Pravsgaard Christensen, Allan Randrup Thomsen

**Affiliations:** ^1^ Department of Immunology and Microbiology, University of Copenhagen, Copenhagen, Denmark; ^2^ Department of Microbiology and Immunology, Emory University, Atlanta, GA, United States

**Keywords:** vaccines, resident memory T cells (Trm), virus’, mucosal surface, CD8 T cell, herd immunity

## Abstract

**Introduction:**

Mucosal immunization eliciting local T-cell memory has been suggested for improved protection against respiratory infections caused by viral variants evading pre-existing antibodies. However, it remains unclear whether T-cell targeted vaccines suffice for prevention of viral transmission and to which extent local immunity is important in this context.

**Methods:**

To study the impact of T-cell vaccination on the course of viral respiratory infection and in particular the capacity to inhibit viral transmission, we used a mouse model involving natural murine parainfluenza infection with a luciferase encoding virus and an adenovirus based nucleoprotein targeting vaccine.

**Results and discussion:**

Prior intranasal immunization inducing strong mucosal CD8+ T cell immunity provided an almost immediate shut-down of the incipient infection and completely inhibited contact based viral spreading. If this first line of defense did not operate, as in parentally immunized mice, recirculating T cells participated in accelerated viral control that reduced the intensity of inter-individual transmission. These observations underscore the importance of pursuing the development of mucosal T-cell inducing vaccines for optimal protection of the individual and inhibition of inter-individual transmission (herd immunity), while at the same time explain why induction of a strong systemic T-cell response may still impact viral transmission.

## Introduction

Being in close and continual contact with the external environment, the respiratory tract is a major portal of entry for a variety of pathogens. Respiratory viruses, such as influenza virus, coronavirus, respiratory syncytial virus (RSV), and parainfluenza virus (PIV), cause mild upper respiratory infections and cold-like symptoms but may also cause bronchitis, pneumonia, and even death, as a result of increased lower respiratory tract involvement. With limited effective prevention or therapy currently, these infections are highly contagious to young children, the elderly and immunocompromised patients, leading to hospitalization and mortality. SARS-CoV-2, the cause of the COVID-19 pandemic, has resulted in over 620 million cases and over 6.5 million deaths worldwide (WHO, 2022). Seasonal influenza epidemics usually occur during winter and can infect up to 20% of the population, depending on which viruses are circulating, and they can cause substantial mortality - up to 650 000 deaths worldwide annually (WHO). Similarly, RSV circulates seasonally and is being increasingly recognized with morbidity and mortality among children and the elderly approaching what is seen with influenza (1). PIV also commonly infects infants and children, and it is still in the absence of effective prevention or therapy for human PIVs illness (CDC).

Given the global burden of viral respiratory infections, there is an urgent need for vaccination to boost protective immune responses. Most current vaccine strategies focus on the induction of neutralizing antibodies which primarily target conformational epitopes on surface proteins and rarely provide substantial cross-protection against viral escape variants (2, 3). In contrast, T cell epitopes consist of short linear amino acid sequences (4) which are widely distributed across proteins, including internal viral components, e.g. viral nucleoprotein, which are much more conserved across variants. Therefore invoking T cell immunity potentially could bring huge advantages to vaccines by offering broad and durable protection. Several studies have shown that specific T cell responses inversely correlate with disease risk and severity (5–10). There are three major subsets of memory T cells as defined by recent studies, which are termed central memory T cells (Tcm), effector memory T cells (Tem), and tissue resident memory T cells (Trm) (11, 12). Trm cells are sessile antigen-primed cells positioned in various non-lymphoid organs, particularly the barrier tissues skin and mucosa, but also in certain internal organs like brain and liver. In the upper and lower respiratory tract, Trm cells have been shown to play a critical role in the early local defense against respiratory infections, such as influenza (13, 14), RSV (15–17), and SARS-CoV-2 (18). The development of a stable population of respiratory tract Trm generally requires local antigen recognition (19–21), whereas conventional parenteral immunization do not suffice to drive substantial local accumulation of these cells. Induction of protective mucosal immunity is more likely to reduce transmission, because viral respiratory infections primarily initiate in the upper respiratory tract, which is also an important source for production of virus and virus containing droplets and aerosol particles to subsequently spread to other individuals.

Adenoviral (Ad) vectors have been extensively investigated and applied for vaccine research (22), and Ad vectors has recently been implemented as platforms for real life vaccines against Ebola (23) and corona viruses (24, 25). These vectors stimulate potent CD8+ T cell responses toward inserted transgene antigen with persistent expression leading to long-lasting immunity (21, 26). Using an Ad encoding influenza virus nucleoprotein, we have previously found that intranasal (i.n.) vaccination elicits a more prolonged protection than systemic vaccination through the footpad (s.c.). However, vaccination by the former route was relatively inefficient in inducing potent systemic T-cell memory as evident by fewer circulating antigen specific memory T cells. In contrast, combined vaccination provided very most robust and durable protection, which highlighted not only the role of resident memory T cells as a critical first line of defense, but also the important back-up role for circulating memory T cells that can expand and migrate to the infected tissue (21, 27).

Despite that a clear association between cell mediated immunity and host protection against severe disease has been demonstrated (5–9), the impact of a rapid T-cell response on transmissibility has only recently been studied, and the potential of using T-cell targeted vaccination to interrupt virus transmission has not been evaluated in detail. Since T cells need to detect and eliminate infected cells, a key question is whether vaccine-induced T-cell immunity may lower viral loads and infectiousness in immunized hosts fast and efficiently enough to prevent inter-individual transmission. Information on this issue is clearly of great significance if the goal is to elicit herd immunity through T-cell targeted vaccination.

Since human influenza viruses and coronaviruses are poorly infectious in mice without adaptation, attempts to establish relevant mouse models for studies of viral transmission have been relatively unsuccessful (28, 29). Related to human PIV, Sendai virus is a natural mouse pathogen that readily transmits between co-housed individuals (30, 31). Furthermore, by using luciferase-expressing challenge virus (rSeV) and a non-invasive *in vivo* imaging system (IVIS), we could - based on earlier observations showing that the intensity of bioluminescence correlates with the extent of viral infection (31) - perform a detailed mapping of the kinetics of the virus infection on an individual basis,. Using this model system we compared the durable protection afforded by systemic vs systemic plus local immunization with an Ad vector encoding Sendai virus nucleoprotein. We found that compared to s.c. vaccination – inducing systemic immunity only -, the combined local and systemic immunity induced by i.n.+s.c. vaccination elicited highly effective and immediate T-cell dependent virus control, which through the release of IFNγ could completely prevent virus transmission and lasted for at least five months. Of substantial interest, we also found that while parenteral vaccination alone failed to provide immediate virus control in the respiratory tract, systemic immunity still significantly reduced onward transmission, albeit not with the same efficiency as that associated with airway immunization.

## Materials and methods

### Mice

Female C57BL/6 mice aged 6-8 weeks were purchased from Taconic Biosciences. Upon arrival, all mice were rested for ≥ 1 week before use. Interferon-gamma deficient (IFNγ -/-), interferon-gamma receptor deficient (IFNγR -/-) and perforin deficient (perforin -/-) mice on the C57BL/6 background were originally obtained from Jackson Laboratory and bred locally in the animal facility at the Faculty of Health and Medical Sciences at the University of Copenhagen. All experiments were conducted in accordance with national Danish guidelines regarding animal experiments as approved by the Danish Animal Experiments Inspectorate, Ministry of Justice (protocol code 2020-15-0201-00585, start date 20200101). The mice were housed in an AAALAC accredited facility in accordance with good animal practice as defined by FELASA.

### Vaccination

Mice were first anesthetized with inhaled isoflurane (Baxter Healthcare Corporation) and immunized intranasally (i.n.) and/or subcutaneously (s.c.) in the right footpad. A replication-deficient adenovirus (Ad) type 5 with deleted E1 and a non-functional E3 gene was used at a dose of 2 × 10^7^ plaque-forming units (PFU) in 30 μl of PBS. Ad expressing Sendai virus nucleoprotein (Ad-SenNP) was administrated i.n.+s.c. or s.c. only. Ad carrying the nucleoprotein from influenza virus strain A/Puerto Rico/8/1934 (Ad-fluNP) was used as sham control and given i.n. and s.c. These adeno vectors were produced and stored as described previously (27).

### Virus challenge and transmission

A luciferase-expressing recombinant Sendai virus (rSeV) was kindly provided by Charlie J. Russell, St. Jude Children’s Research Hospital (31, 32). For Sendai virus challenge, rSeV was used at a dose of 1500 PFU in 30 μl PBS and administrated i.n. after isoflurane anesthesia. For transmission studies, index mice were i.n. infected with 1500 PFU rSeV and one day later each of these were transferred to a cage with four naïve mice; mice were co-housed until the experiment was terminated.

### Quantification of Sendai virus

Mice were anesthetized by intraperitoneal (i.p.) injection with avertin (2,2,2 tribromoethanol in 2-methyl-2-butanol, 250 mg/kg, Alfa Aesar) and exsanguinated. For nasal flush, a catheter was gently inserted into the trachea, 0.5 ml 1% BSA in PBS was flushed from the trachea to the nasopharynx, and the liquid coming out from the nares was collected. Lungs were homogenized in 1% FBS in PBS using sterile sand, mortar, and pestle to obtain a 10% weight/volume suspension. Homogenates were then clarified by centrifugation at 600 × g, for 15 minutes at 4°C. The nasal flush and lung sample supernatants were stored at -80°C until use.

Vero cells grown in DMEM 1965 with NaHCO_3_ supplemented with 10% FBS, 200 IU/ml Penicillin, 50 μg/ml Streptomycin, 2 mM L-glutamine, 1% Sodium Pyruvate at 37°C, 5% CO_2_ were used for Sendai plaque assay. 1 × 10^5^ cells were seeded in 24-well plates and the following day, cells were washed with PBS and then incubated with 200 μl of tenfold diluted virus samples in serum free media with 5 units/ml TPCK Trypsin (Sigma-Aldrich) for 1 hour at 37 °C and 5% CO_2_. Virus samples were then gently removed, and an overlay media prepared with 50% 2 × eagle’s minimal essential medium (MEM) with NaHCO_3_, 0.2% BSA, 200 IU/ml Penicillin, 50 μg/ml Streptomycin, 2 mM L-glutamine, 1% Sodium Pyruvate, 50% methylcellulose (1.8% 400cp), 5 units/ml TPCK Trypsin was added to the cells. After incubation for 4 days at 37 °C, 5% CO_2_, overlay was removed and cells were fixed with 4% formaldehyde in PBS for 15 minutes, washed with PBS, and stained with 0.1% crystal violet (Sigma-Aldrich) in PBS for 15 minutes at room temperature. PFU/ml nasal flush and PFU/g lung tissue were calculated accordingly:


dilution factor×average number of plaque/well×5=PFU/ml

dilution factor×average number of plaque/well×50=PFU/g

### *In vivo* imaging system

The rSeV infection of challenged or co-housed mice was quantified by bioluminescence measurement. Every 24 hours mice were injected i.p. with 3 mg luciferin substrate (PerkinElmer) in PBS. After 15 minutes, mice were anesthetized using inhaled isoflurane through a 5-port manifold, placed in supine positions, scanned for bioluminescence by the IVIS CCD camera (Caliper LifeSciences) using auto-exposure and a 23 cm imaging field of view (FOV). Images were analyzed with Living Image 4.3.1 software (PerkinElmer). To quantify bioluminescence, square regions of interest (ROI) were defined manually including the upper and lower respiratory tract (nasopharynx, trachea and lungs) of each animal, quantified and expressed as “Avg Radiance” - numbers of photons emitted per unit time from a defined area (photons per second per square centimeter per steradian, p/s/cm²/sr). Bioluminescence curves were graphed over time, and the area under the curve (AUC) was calculated using GraphPad Prism with one as the baseline.

### T cell effector functions depletion and blockade

For local CD8 T cells depletion, mice were treated with monoclonal α-CD8a antibody (clone YTS 169.4, BioXcell) 1 day before challenge using 5 μg i.n. For systemic CD8 T cell depletion, mice were treated 1 day before challenge with 200 μg i.p., followed by 100 μg i.p. on day 1 and day 4 post challenge. Fingolimod (FTY720, Sigma-Aldrich) was dissolved to 2.5 mg/L in drinking water and given from day 3 prior to challenge, this strategy has previously been found to prevent CD8+ T cell recirculation (33).

### Single cell preparation and flow cytometry

To isolate tissue-positioned lymphocytes, mice were intravenously injected with 1.5 μg PE-CF594-conjugated α-CD3e (clone 145-2C11) fluorophore-conjugated antibody in 200 μl PBS in the tail vein. Five minutes later, mice were anesthetized using avertin and exsanguated. For bronchoalveolar lavage (BAL) sampling, the trachea was exposed and a small incision was made for inserting a catheter. The lungs were flushed 5 times with 1 ml cold Hanks BSS medium, and the lavage fluid was collected. For single cell preparation of mediastinal lymph node (MLN) and spleen, these organs were aseptically removed, mechanically dissociated and mashed through a 70 µm nylon cell-strainer (BD falcon). Lungs were harvested and digested with 5 g/L Collagenase D (Roche) and 2 × 10^6^ units/L DNAse (Sigma) for 30 minutes, 37°C, and lymphocytes were enriched by 40%/80% Percoll gradient centrifugation. Cell counts were done by an automated cell counter Countess (Invitrogen).

Cells were stained with NP324-332/Kb (FAPGNYPAL) tetramers conjugated to allophycocyanin (APC) for 1 hour, dark at room temperature. Subsequently cells were washed twice with PBS and stained with ZombieNIR (Biolegend) and fluorochrome conjugated antibodies: BV785 conjugated α-CD8a (clone 53-6.7), FITC conjugated α-CD44 (clone IM7), PE conjugated α-CD69 (clone H1.2F3), and BV605 conjugated α-CD103 (clone 2E7) in PBS for 20 minutes, dark on ice. Samples were analyzed by LSRFortessa analyzer (BD Biosciences) with data analysis conducted using FlowJo software version 10 (TreeStar); representative plots detailing the gating strategy are depicted in **Supplementary Figure 1**. All antibodies were purchased from Biolegend and tetramers were kindly provided by Søren Buus of this institute.

### Statistical analysis

Statistical analysis was carried out using Excel and GraphPad Prism. For comparisons of more than two groups, one-way ANOVA followed by Tukey’s multiple comparisons test, or Kruskal-Wallis test followed by Dunn’s multiple comparisons test. Only if groups were found to differ significantly, statistical comparison between two groups was assessed using the Mann-Whitney rank sum test (two-tailed). Levels of statistical significance is represented as: ns (not significant), *(P < 0.05), **(P < 0.01), ***(P < 0.001), ****(P < 0.0001).

## Results

### Combined intranasal and systemic immunization with Ad5 encoding Sendai nucleoprotein induces long standing antigen-specific CD8+ T cell memory in the airways

**Figure 1:**
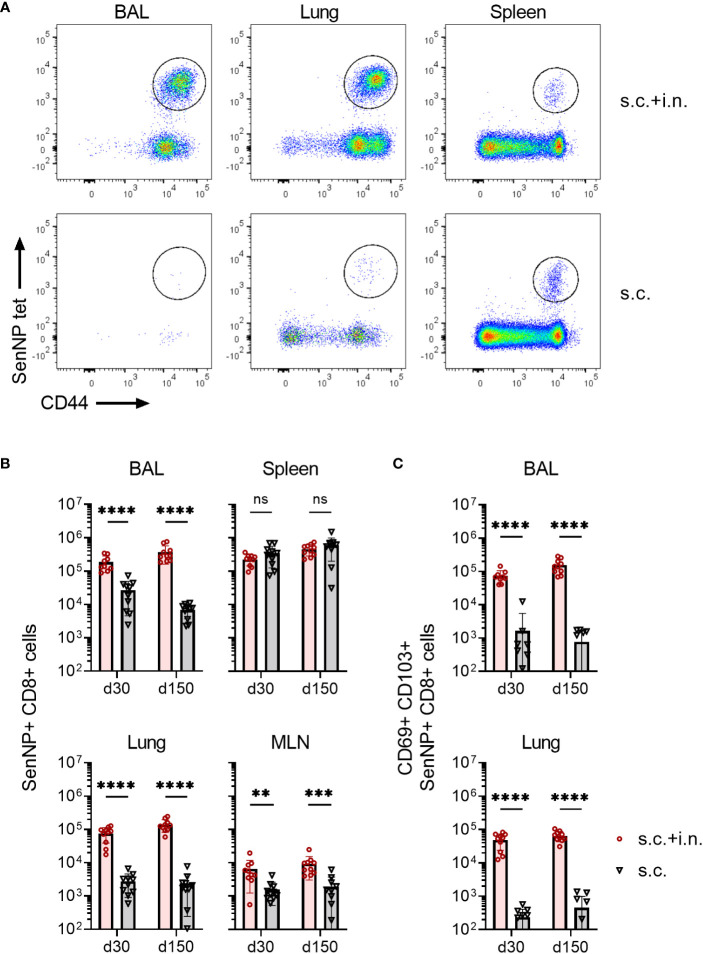
Magnitude and localization of antigen-specific memory CD8+ T cells elicited by Ad5 encoding Sendai nucleoprotein. C57BL/6 mice were immunized with Ad-SenNP s.c.+i.n. or s.c. Thirty and 150 days later (labeled as d30, d150), mice were given flourochrome labelled anti CD3 i.v. and spleen, MLN, lungs, and BAL were isolated for tetramer analysis. **(A)** Representative plots at d150 and **(B)** absolute numbers of SenNP+CD8+ T cells. **(C)** Absolute numbers of SenNP+CD8+ T cells that co-expressed CD69 and CD103. In B and C, each dot represents one animal and bars represent means with SD, based on groups of 10 animals. Statistical significance was determined using Mann-Whitney test; ns (not significant), **(P < 0.01), ***(P < 0.001), ****(P < 0.0001).

First, we evaluated the immunogenicity of the chosen vector system, and how the route of immunization influenced the magnitude and localization of the elicited CD8+ T cell response. To this end, C57BL/6 mice were immunized with an adenovector vaccine expressing the Sendai virus nucleoprotein (Ad-SenNP) either in the footpad (s.c.) only or by application of the vaccine s.c. as well as intranasally. Thirty and 150 days later (d30, d150), antigen-specific CD8+ T cells was quantified in bronchoalveolar lavage (BAL), lungs, mediastinal lymph nodes (MLN), and spleens using flow cytometry. Intravascular staining with flourochrome labelled anti-CD3 was used to differentiate circulating (anti-CD3+) and tissue-positioned (anti-CD3-) T cells in the respiratory system. Antigen-specific memory CD8+ T cells were determined by tetramer staining of CD8+ T cells targeting a dominant Kb-restricted peptide epitope of the Sendai nucleoprotein (SenNP), together with T cell activation marker CD44 (**Figure 1A**).

Robust numbers of SenNP-specific CD8+ memory T cells emerged in the spleen following either systemic or combined routes of Ad-SenNP vaccination (**Figure 1B**). In addition to a systemic response, high numbers of these cells were more efficiently induced in the MLN, BAL, and lungs by combined (s.c.+i.n.) compared to s.c. vaccination. Notably, these BAL and lung located antigen specific CD8+ memory T cells co-expressed CD69 and CD103 to a substantially higher degree in s.c.+i.n. animals, suggesting tissue resident properties, whereas s.c. immunization established mainly a systemic pool of memory CD8+ T cells (**Figure 1C**). The pronounced SenNP-specific CD8+ memory T cell response was seen locally and systemically at day 30 and lasted for at least 150 days (**Figures 1B, C**).

### Protective capacity of local versus systemic immunity against viral challenge

To evaluate the protection afforded by having an expanded local CD8+ T cell population versus systemic immunity only, mice were immunized with the Ad-SenNP vaccine via s.c.+ i.n. or s.c. To control for a possible impact of trained immunity, a similar Ad5 vector encoding an unrelated nucleoprotein from influenza virus was used as a sham vaccine and this was also given s.c.+i.n.; age-matched unvaccinated mice served as negative controls. Thirty or 150 days after vaccination mice were challenged with 1500 PFU of rSeV encoding the gene for firefly luciferase and differences in the dynamics of infection in vaccinated versus control mice as revealed in the pattern of bioluminescence was followed for 10 days.

**Figure 2:**
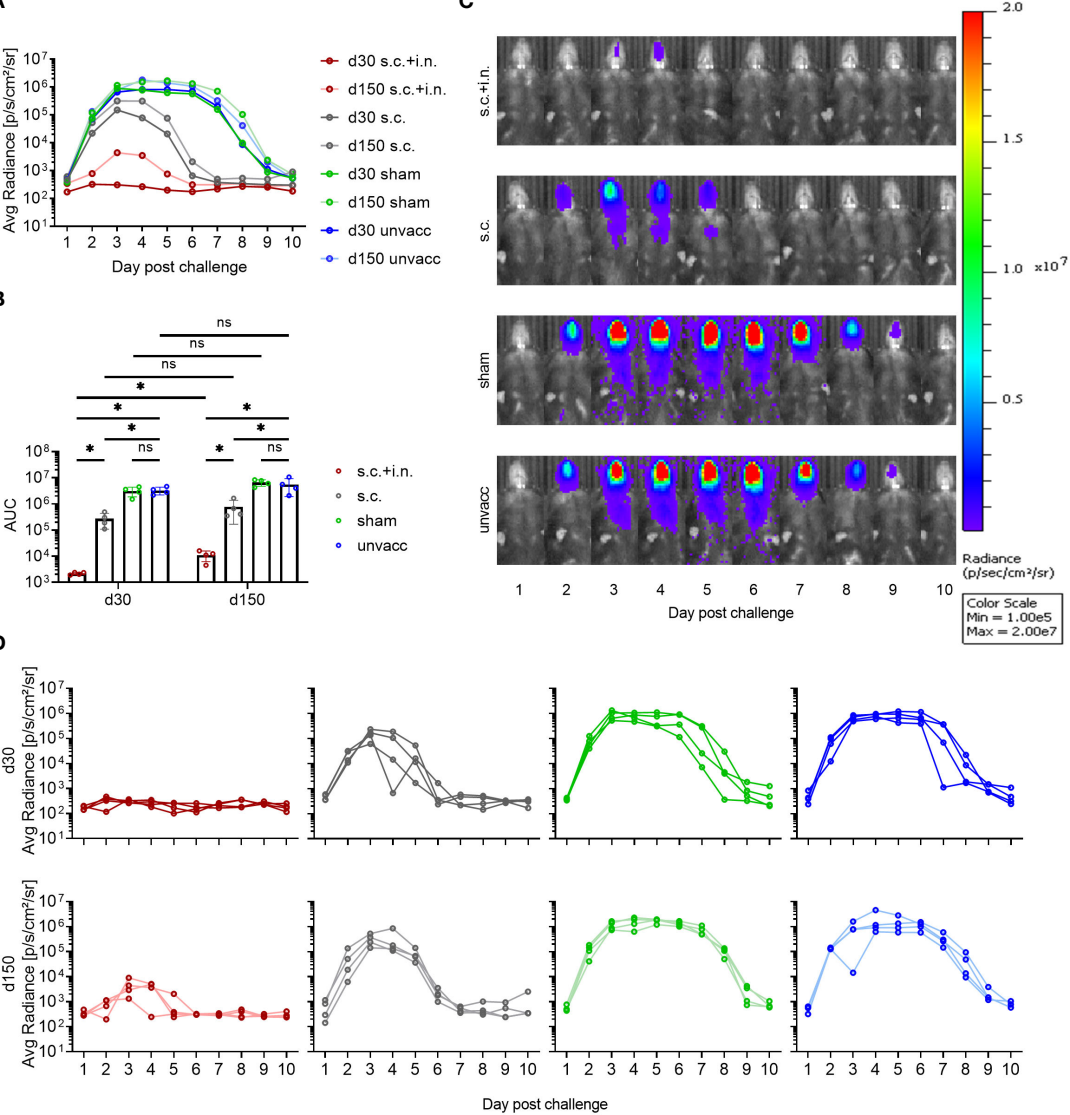
Protective capacity of local versus systemic immunity against viral challenge. C57BL/6 mice were immunized with Ad-SenNP s.c.+i.n., Ad-SenNP s.c., or Ad-fluNP s.c.+i.n. (sham control), with unvaccinated mice as controls (unvacc). Thirty and 150 days later (d30, d150), all mice were challenged i.n. with 1500 PFU rSeV expressing luciferase. Each curve represents **(A)** the average or **(D)** the individual bioluminescence of 4 mice per group. **(B)** Bars represent the mean AUC of bioluminescence with SD error bars and individual data points. Statistical significance was determined using Mann-Whitney test; ns (not significant), *(P < 0.05). **(C)** Representative images of a single mouse from each group at d150 were displayed as radiance on a colorimetric scale ranging from 1.0E5 (blue) to 2.0E7 (red) photons/s/cm2/steradian.

Regardless of time after vaccination, mice previously subjected to combined local and systemic immunization with Ad-SenNP (s.c.+i.n.) had an absolute advantage in controlling the degree of viral infection, and this effect was obvious from the very beginning (**Figures 2A, D**). There was no detectable replication in the lung, and very little if any replication in the upper airways (see **Figure 2C** for results in mice challenged 150 days after vaccination). In contrast, systemic immunity (induced by s.c. immunization only) did not play a significant role in the first 2 days but began to impact viral replication around day 3 post challenge, and an accelerated decline in bioluminescence was observed. Lacking specific immunity prior to challenge, sham vaccinated and unvaccinated controls continued to support an increasing bioluminescence that peaked at 1.0E6 p/s/cm2/sr. This was higher than that following s.c. vaccination, and unlike the situation in mice with pre-established systemic immunity, the level of infection remained high for a week (**Figures 2A, C, D**).

The remarkable protection induced by combined local or systemic immunization with the Ad-SenNP vaccine had a durability of at least 5 months. However, while there was no significant difference in the pattern of bioluminescence between 30 and 150 days post vaccination in any other group, the protection afforded by combined vaccination seemed to wane slightly over time, resulting in a slightly higher peak bioluminescence as well as a higher AUC at d150 compared to d30 post vaccination (**Figures 2A, B**). Nevertheless, very efficient protection was still observed at this time point.

### Role of CD8+ T cells in vaccine induced protection against viral infection

The protective mechanisms elicited by Ad-SenNP were further assessed using CD8 depletion with monoclonal antibodies and Fingolimod (FTY720) treatment. Animals were either combined or s.c. vaccinated, challenged at day 30, and infection induced bioluminescence was measured as described previously.

**Figure 3:**
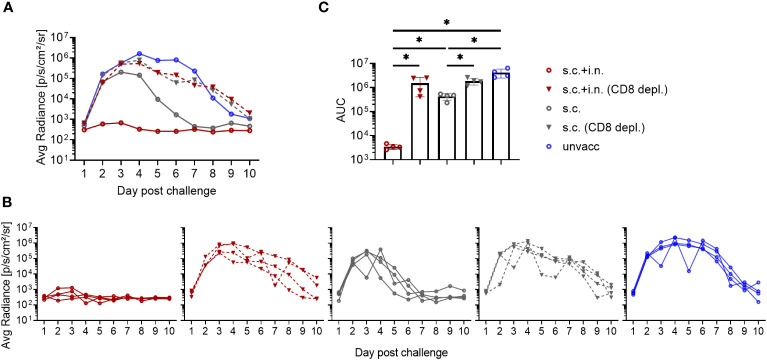
Role of CD8+ T cells in vaccine induced protection against viral infection. C57BL/6 mice were immunized with Ad-SenNP s.c.+i.n. or s.c. Thirty days later these mice, together with unvaccinated controls (unvacc) received an i.n. challenge of 1500 PFU rSeV. Part of the vaccinated mice were subjected to *in vivo* CD8+ T cell depletion as described in Materials and Methods. Each curve represents **(A)** the average or **(B)** the individual bioluminescence of 4 mice per group. **(C)** Bars represent the mean AUC of bioluminescence with SD error bars and individual data points. Statistical significance was determined using Mann-Whitney test; ns (not significant), *(P < 0.05).

The infection in s.c.+ i.n., s.c., and unvaccinated mice repeatedly showed distinct dynamics and significantly different AUC regarding bioluminescence, correlating to the level of induced local or systemic immunity. When CD8+ T cells were depleted, similar lack of immunity was seen in both groups of vaccinated mice. The robust protection resulting from added local immunization was fully abrogated, and the impact of systemic immunity seen from day 3 and onwards was also greatly affected, leading to delayed virus clearance and markedly higher AUC in depleted mice (**Figure 3**). Thus, a central role of CD8+ T cells was evident in both the local and systemic immunity against rSeV infection.

**Figure 4:**
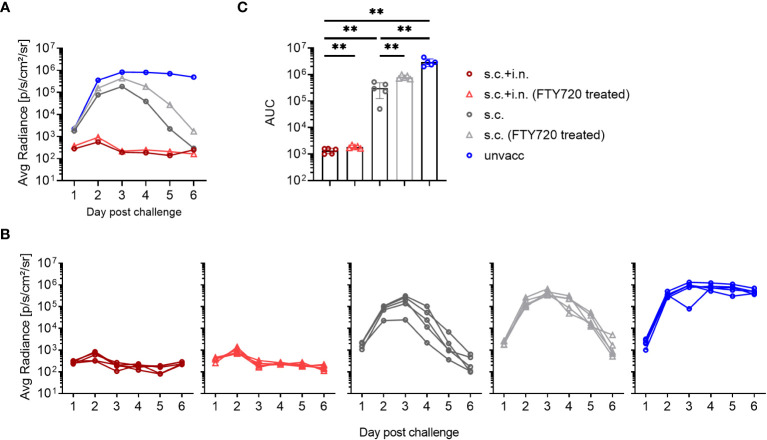
Role of recirculating lymphocytes. C57BL/6 were immunized with Ad-SenNP s.c.+i.n. or s.c. Thirty days later, these mice together with unvaccinated controls (unvacc), were challenged i.n. with 1500 PFU rSeV. Part of the vaccinated mice were given Fingolimod (FTY720) as described in Materials and Methods. Each curve represents **(A)** the average or **(B)** the individual bioluminescence of 5 mice per group. **(C)** Bars represent the mean AUC of bioluminescence with SD error bars and individual data points. Statistical significance was determined using Mann-Whitney test; ns (not significant), **(P < 0.01).

To gauge the relevance of recirculating cells, FTY720 was used to prevent lymphocyte egress from secondary lymphoid tissues. We found that FTY720 treatment delayed the protective response in s.c. vaccinated mice, with slightly higher bioluminescence levels at early times post infection, which became more pronounced during days 3-6 post challenge, resulting in a higher average AUC than that observed in s.c. vaccinated mice without treatment. On the other hand, FTY720 had little, if any effect on virus control and marginally increased the AUC of bioluminescence in combined vaccinated mice, consistent with the idea that tissue resident cells played the major role in these mice and that secondary recruitment of circulating effector T cells played little if any role in this case (**Figure 4**).

### CD8+ T cells mediate early resistance to viral challenge primarily through release of IFNγ

To determine the immunological effector mechanisms of the immediate protection against reinfection, IFNγ -/-, or perforin -/- mice were included. In this case mice subjected to combined vaccination were challenged 30 days later, and bioluminescence was measured for the first 3 days after challenge.

**Figure 5:**
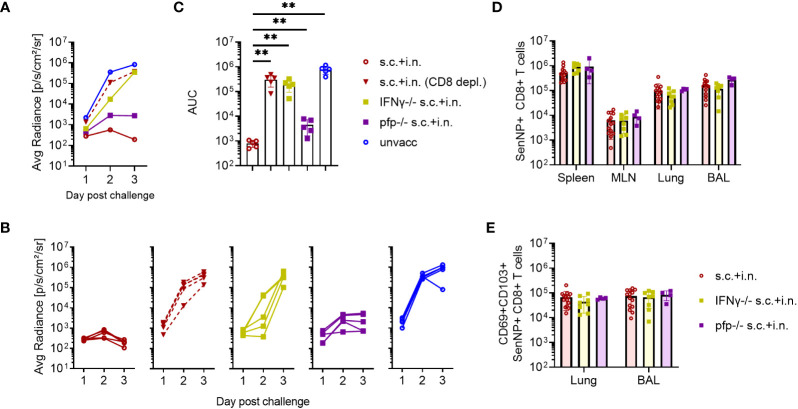
CD8+ T cells and effector molecules mediate resistance to viral challenge. C57BL/6 wild type, IFNγ -/-, and perforin (pfp) -/- mice were immunized with Ad-SenNP s.c.+i.n. Thirty days later these mice, together with unvaccinated controls (unvacc, the same as in **Figure 4**)), were i.n. challenge with 1500 PFU rSeV. A group of vaccinated (s.c.+i.n.) C57BL/6 mice depleted of CD8+ T cells as previously described were included for comparison. Each curve represents **(A)** the average or **(B)** the individual bioluminescence of 5 mice per group. **(C)** Bars represent the mean AUC of bioluminescence with SD error bars and individual data points. Statistical significance was determined using Mann-Whitney test; ns (not significant), **(P < 0.01). C57BL/6 wild type, IFNγ -/-, pfp -/- mice were immunized s.c.+i.n. and thirty days later, spleen, MLN, lungs, and BAL were isolated for tetramer analysis of **(D)** absolute number of SenNP+CD8+ T cells and **(E)** absolute numbers of SenNP+CD8+ T cells in the airways that co-expressed CD69 and CD103. Each dot represents one animal and bars represent means with SD, based on a group of 4-14 animals. Statistical significance was determined using Mann-Whitney test, and no significant difference was observed in **(D, E)**.

Both the lack of IFNγ or perforin molecule impacted the early virus control elicited by combined vaccination, but to a quite different extent. Thus, the effect of perforin was rather limited, while the infection in IFNγ -/- and CD8+ T-cell depleted mice continued to develop, indicating substantially reduced immunity from the combined vaccination in these mice (**Figures 5A–C**). Matching results were obtained in IFNγR -/- mice (data not shown).

Interestingly, in a follow-up analysis where we studied mechanisms of virus control at latter stages (**Supplementary Figure 3**), we saw that although virus control was markedly delayed in IFNγ -/- mice, virus replication was eventually shut down also in these mice. In the light of the prolonged impact of CD8 depletion revealed in **Figures 3**, **5**, this observation points to the relevance of additional vaccine-induced CD8+ T-cell dependent effector mechanism in the later phases of infection. Which these are is not presently clear, at no point did we see a marked impact of lacking perforin.

To ascertain that the observed reduction in protection did not reflect differences in the number or localization of primed SenNP-specific CD8+ T cells in vaccinated gene targeted mice, we compared the magnitude and distribution of memory CD8+ T cells in these mice to that in similarly vaccinated wild type mice. Importantly, similar numbers of SenNP-specific CD8+ memory T cells were recovered from the circulation and local tissues of all types of vaccinated mice, indicating that the observed reduction in protection directly reflected reduced effector capacity of primed CD8+ T cells in the gene targeted mice (**Figures 5D, E**).

### Transmission capacity and local viral titers are differentially impacted by local and systemic immunity

**Figure 6:**
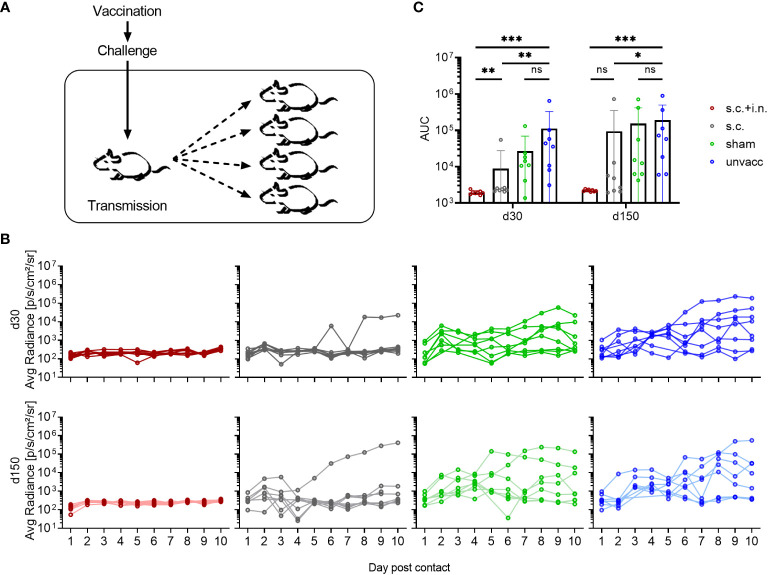
Transmission capacity is impacted by local and systemic immunity. **(A)** C57BL/6 mice immunized with Ad-SenNP s.c.+i.n., Ad-SenNP s.c., or Ad-fluNP s.c.+i.n. (sham control), and unvaccinated controls were used as index mice. Thirty and 150 days later (d30, d150), each of these animals was challenged i.n. with 1500 PFU rSeV and one day later co-housed with four naïve mice. **(B)** Curves represent bioluminescence of 8 contact mice per group. **(C)** Bars represent the mean AUC of bioluminescence with SD error bars and individual data points. Statistical significance was determined using Mann-Whitney test; ns (not significant), *(P < 0.05), **(P < 0.01), ***(P < 0.001).

Since prior vaccination clearly reduced viral replication following challenge, the next step was to assess whether the subsequent transmission was impacted by local and systemic immunity. Therefore, co-housing experiments were set up, with the four groups of differentially treated mice (referred to as index mice): vaccinated with Ad-SenNP s.c.+i.n. or s.c. only, Ad-fluNP s.c.+i.n (sham control), and unvaccinated. Each of the index mice were infected with rSeV 30 or 150 days after vaccination, and 1 day later co-housed with 4 unvaccinated naïve mice, referred to as contact mice (**Figure 6A**). The viral spreading, which was manifested by transmission to the contact mice, was followed by bioluminescence measurements for 10 days post contact.

No bioluminescence was detected in the contact mice co-housed with s.c.+i.n vaccinated index mouse, neither early nor late after vaccination. Evidence of limited early transmission was observed in cages with s.c. vaccinated index mice, particularly when challenged 150 days after vaccination. In this case detectable bioluminescence in contact mice tended to decline by day 3-4 and mostly became absent thereafter, while full blown infection was noted only in a few of the contact mice. This pattern is indicative of transmission, but at low-level intensity. Following co-housing with challenged sham vaccinated and unvaccinated index mice, early transmission was noted in the majority of cases, and in a high proportion of these mice evidence of prolonged infection was observed. The sham and unvaccinated index mice transmitted the virus to a similar extent, while the AUCs of bioluminescence in the contact mice co-housed with s.c.+i.n. and s.c. index mice was significantly decreased. All index mice seemed to retain a similar transmission capacity at days 30 and 150 post vaccination, based on infection of contact mice (**Figures 6B, C**).

**Figure 7:**
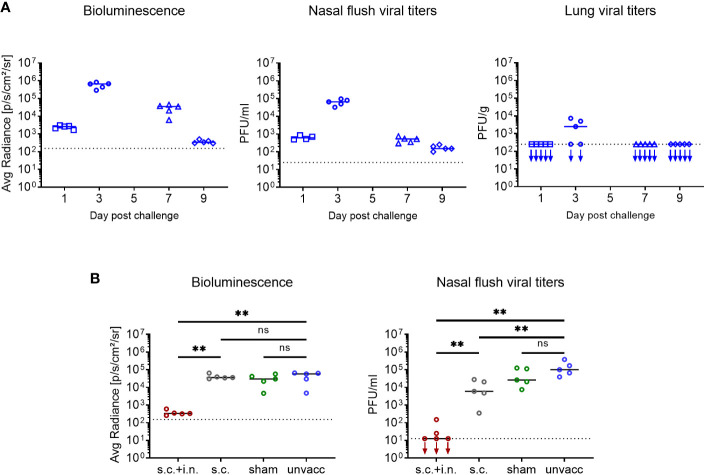
Correlation between bioluminescence and organ virus titers. **(A)** C57BL/6 mice were infected i.n. with 1500 PFU rSeV, and on days 1, 3, 7, 9 post challenge measured for bioluminescence and viral titers in nasal flush and lungs. **(B)** C57BL/6 mice were immunized with Ad-SenNP s.c.+i.n., Ad-SenNP s.c., or Ad-fluNP s.c.+i.n. (sham control). Thirty days later these mice and unvaccinated controls were infected i.n. with 1500 PFU rSeV, on day 3 post challenge measured for bioluminescence and viral titers in nasal flush. Each dot represents one animal and lines represent group medians, with 4 or 5 mice per group. The dotted line indicates the mean bioluminescence measured on 2 animals for several days without rSeV infection, or the detection limit of viral titers. Statistical significance was determined using Mann-Whitney test; ns (not significant), **(P < 0.01).

Considering that it was a great surprise that prior local immunization with a CD8+ T cell targeting vaccine caused almost sterile immunity – for a comparison with the natural immunity following Sendai infection inducing both humoral and cell mediated immunity, see **Supplementary Figure 2** - and zero transmission, we wanted to validate our methodology and directly measure the local viral replication. In naïve mice lacking pre-existing immunity, both bioluminescence and viral titers peaked 3 days after infection. By day 7, the nasal flush samples contained much less virus. The bioluminescence signal was also substantially reduced at this time-point, albeit not to quite the same degree, probably reflecting that luciferase was eliminated more slowly than the viral infection per se. Viral titers in the lungs were mostly below the level of detectability (**Figure 7A**). Following this validation of our experimental approach, mice were vaccinated either s.c. +i.n. or s.c. For control, other groups of mice were subjected to combined vaccination with an irrelevant vaccine or left untreated. Thirty days later all mice were challenged i.n., and viral titers in nasal flush was determined on day 3 post challenge. Similar to what had been observed previously, combined vaccination was able to significantly restrain viral replication, as both bioluminescence and viral titers were greatly reduced, and in fact, in three out of five mice we could not detect infectious virus in the nasal flush. The s.c. vaccinated mice showed comparable bioluminescence but slightly lower viral titers, compared to the unvaccinated controls. And the sham and unvaccinated mice did not significantly differ from each other in terms of neither bioluminescence nor viral titers (**Figure 7B**).

### Role of CD8+ T cells in the immunological control of inter-individual virus transmission

**Figure 8:**
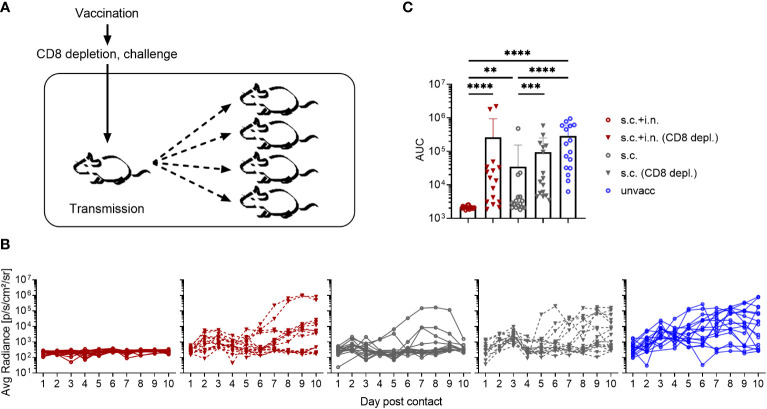
Role of CD8+ T cells in preventing virus transmission. **(A)** C57BL/6 mice were immunized with Ad-SenNP s.c.+i.n. or s.c. Thirty days later, each of these and unvaccinated index mice were infected i.n. with 1500 PFU rSeV and one day later co-housed with four naïve animals. Part of the vaccinated index animals were depleted of CD8+T cells as previously described. **(B)** Curves represent bioluminescence of 16 contact mice per group. **(C)** Bars represent the mean AUC of bioluminescence with SD error bars and individual data points. Statistical significance was determined using Mann-Whitney test; ns (not significant), **(P < 0.01), ***(P < 0.001), ****(P < 0.0001).

Finally, to investigate the role of CD8+ T cells in controlling virus transmission after vaccination, mice immunized with Ad-SenNP s.c.+i.n. or s.c. as well as unvaccinated controls were used as index mice in co-housing experiments. Thirty days post vaccination, part of the vaccinated mice were depleted of their CD8+ T cells and all the mice were challenged i.n. and co-housed with naïve animals as described previously (**Figure 8A**).

Again the transmission capacity of s.c.+i.n., s.c., and unvaccinated index mice were significantly different from one another, correlating with the route of immunization. Compared to the absence of detectable transmission from combined vaccinated mice with an intact CD8 population, clear transmission from such immunized mice occurred following CD8+ T cell depletion. Already in the early phase - day 1-2 of cohousing - the lack of control of transmission by resident CD8+ T cells was noteworthy, with elevated bioluminescence in contact mice co-housed with s.c.+i.n. (CD8 depleted) index mice. Again, s.c. vaccinated mice transmitted more than matched s.c.+i.n. index mice. Co-housing with s.c. immunized index mice depleted of CD8+ T cells lead to a further increase in the bioluminescence, distinguishing these contact mice from those co-housed with s.c. immunized index mice without depletion as early as day 3 post contact. Moreover, when we looked at the bioluminescence in contact mice at a later stage during day 5-10 post co-housing, we observed evidence of more frequent, prior substantial viral transmission as well as a higher AUC of bioluminescence in the mice co-housed with s.c. (CD8+ T-cell depleted) mice (**Figures 8B, C**).

## Discussion

The current SARS-CoV-2 pandemic has highlighted some key unknowns regarding vaccine induced prevention of respiratory tract infections. One relevant question is, how efficiently does parenteral immunization, eliciting a potent systemic T-cell response, impact the capacity of the immunized host to transmit the infection? Or to phrase it differently, can we rely on systemic vaccination at all for induction of herd immunity? Results from animal studies have clearly emphasized the importance of local immunization in order to obtain optimal immune mediated protection in the airways, whereas systemic immunity fails to provide a similar first line of defense (13, 27). When the new corona vaccines were introduced, authorities recommended vaccination arguing that you should get vaccinated not only for your own sake, but also to protect risk groups such as the elderly. This communication strategy implied a significant effect on viral transmission in addition to protection of the individual by systemic vaccinations. While epidemiological studies suggest that this may have been partly true with the earliest SARS-CoV-2 variants of relatively low infectiousness, this view point is more difficult to uphold with circulation of highly infectious variants of the omicron lineage (34–37). Given that T cells primarily target internal proteins, which are not subject to ongoing selection by neutralizing antibodies, it would be logical to target e.g. viral nucleoprotein in a new generation of more robust vaccines with increased breadth of antiviral protection against antibody selected escape variants. However, introducing such vaccines requires prior knowledge about the efficiency of the elicited T cell responses not only in protecting the vaccinated individual, but similar to preformed antibodies also to significantly reduce inter-individual transmission. This perhaps represent the most important uncertainty as T cells unlike antibodies are only effective once an infection is already established.

To address these issues, we chose to use murine infection with Sendai virus as our model system. Unlike SARS-CoV-2 and influenza, this is a natural mouse pathogen that easily transmits in mouse colonies. By comparing systemic immunization with combined local and systemic immunization, we could confirm that local immunization in the airways very significantly improved the capacity to resist subsequent challenge with live virus. Our vaccine expressed only the NP gene from the Sendai challenge virus, and thus the response was strongly biased towards a T-cell response (38, 39), as confirmed by the observed impact of CD8 depletion. Quite surprisingly and somewhat counterintuitively, we could conclude that CD8+ T-cell immunity under the right conditions could provide nearly sterile immunity. Obviously, incipient infection must take place in the vaccinated mice, but it borders on the level of detection, and most importantly, no inter-individual transmission is seen from mice with local immunization. Our results also support the importance of local residency as a critical first line of protection, since blocking of T cell circulation using FTY720 does not reduce early protection. The speed and efficiency with which local T cells appear to shut-down the incipient infection even in mice challenged with a relatively high dose, point to a primary role of an antiviral activity based on a diffusible factor. Consistent with this assumption, analysis of relevant gene knock-out mice demonstrates that the early virus control relies heavily on the production of IFNγ, while the importance of perforin, requiring a one-on-one interaction with each infected cell, is very limited at this early stage. Regarding the interpretation of these findings, it should be emphasized that the initial course of infection was identical in unvaccinated knock-out and wild type mice (**Supplementary Figure 3**). Moreover, analysis of the number and distribution of NP-specific CD8+ T cells in the vaccinated knock-out mice revealed no differences in immune cell numbers or recruitment to the airways compared to wild type mice prior to challenge. Thus it is most likely the impairment of the targeted effector function that explains the differences in the observed early virus control. Notably, for the s.c. immunized mice treatment with FTY720 tended to delay virus clearance, suggesting that recruitment of circulating cells play a role, in case the first line of virus control is insufficient. This is in complete accordance with the model for the secondary anti-flu response previously proposed by D. Woodland et al (40).

Regarding the capacity of CD8+ T cells to inhibit viral transmission, we made two important observations. While the importance of local immunization for optimal T-cell mediated control of a viral challenge in itself is unsurprising, the magnitude of the impact resulting from local priming is remarkable: viral replication was hardly detectable. Thus, our results highlight the potential of a strong forward CD8+ T-cell defense to control early viral replication to a degree which almost matches that of preexisting antibodies. Under these conditions it is hardly surprising that subsequent viral transmission is prevented. Thus, co-housing of naïve contact mice with challenged index mice previously subjected to mucosal immunization revealed no detectable infection of the former. However, even in the case of mice vaccinated only s.c., and with no reduction of early viral replication following viral challenge, we still found substantially reduced transmission. Thus, for most of naïve cage mates co-housed with s.c. vaccinated, virus challenged index mice, we saw evidence of limited viral take and only a few developed a full-blown infection. This is unlike the situation in cage mates of virus-challenged sham or unvaccinated index mice. This difference in transmission capacity suggests that the amount of infectious virus released from the challenged parenterally immunized index mice is substantially reduced, despite an almost unimpaired viral replication in the respiratory tract during the first few days of infection. We find this observation to be important as it could suggest that it is perhaps not so much the peak viral load as the longevity of productive infection or a combination of both, which matters most for viral transmission. In this context it should be noted that mice immunized only parentally, still have a distinct population of memory T-cells present in their lungs, which could modify the course of infection. If our findings can be generalized, this holds clear implications for how we should look upon the potential of Covid-19 vaccines to induce effective herd immunity. Evaluation of viral loads in vaccinated human patients with SARS-CoV-2 break-through infections have revealed a pattern strikingly similar to that in parentally immunized mice, with evidence pointing to almost full-blown viral replication in the first few days of infection followed by an accelerated clearance over the next days (41). If therefore, our findings can be directly translated to respiratory tract infections in humans, there is still reason to assume that strong systemic immunity may reduce transmission of viral respiratory tract infections to some degree, although there is clearly a substantial advantage to be gained, if local immunization could be implemented for the next generation of Covid-19 vaccines.

In conclusion, our results have revealed an almost immediate and highly efficient capacity of local antiviral T cells to abort viral infection of the respiratory tract. This supports the feasibility of using T-cell targeted vaccines not only to efficiently control severe viral infection of the respiratory tract, but also as a means to prevent transmission/induce herd immunity. However, for this defense to be most efficient, local immunization leading to elicitation of airway Trm cells is important. Thus our findings emphasizes the need for mucosal vaccines. Nevertheless, even in the absence of local immunization, strong systemic T cell immunity may suffice to accelerate virus clearance and cause some reduction in inter-individual transmission.

## Data availability statement

The raw data supporting the conclusions of this article will be made available by the authors, without undue reservation.

## Ethics statement

The animal study was approved by Danish Animal Experiments Inspectorate, Ministry of Justice (protocol code 2020-15-0201-00585, start date 20200101). The study was conducted in accordance with the local legislation and institutional requirements.

## Author contributions

JZ: Data curation, Formal Analysis, Investigation, Writing – original draft, Writing – review & editing. IU: Conceptualization, Data curation, Formal Analysis, Investigation, Writing – review & editing. JK: Conceptualization, Writing – review & editing. JC: Conceptualization, Supervision, Writing – review & editing. AT: Conceptualization, Investigation, Supervision, Writing – original draft, Writing – review & editing.

## Supplementary material

Supplementary Figure 1 Gating strategy. Lymphocytes were first gated on a forward area and side area plot. After gating single cells on a forward area and height plot and a side area and height plot, live cells were identified with ZombieNIR. Intravascular staining was used to discriminate circulating (i.v. CD3+) and resident (i.v. CD3-) cells in the lungs, BAL and MLN. SenNP+CD44+CD8+ cells were defined as antigen-specific (tetramer+) memory T cells and were then gated into a new plot with CD69 and CD103.

Supplementary Figure 2 Ad-SenNP vaccine efficacy compared to natural immunity. C57BL/6 mice were immunized with Ad-SenNP s.c.+i.n., or rSeV i.n. 30 days ago, together with unvaccinated controls, followed by 1500 PFU rSeV i.n. challenge. Each curve represents **(A)** the average or **(B)** the individual bioluminescence of 8 mice per group. **(C)** Bars represent the mean AUC of bioluminescence with SD error bars and individual data points. Statistical significance was determined using Mann-Whitney test; ns (not significant), ***(P < 0.001).

Supplementary Figure 3 Role of CD8 effector molecules in virus control. C57BL/6 wild type, IFNγ -/-, and perforin (pfp) -/- mice were immunized with Ad-SenNP s.c.+i.n. Thirty days later these mice, together with unvaccinated (unvacc) C57BL/6 wild type, IFNγ -/-, and pfp -/- mice, were i.n. challenge with 1500 PFU rSeV. Each curve represents **(A)** the average or **(B)** the individual bioluminescence of 4-5 mice per group. **(C)** Bars represent the mean AUC of bioluminescence with SD error bars and individual data points. Statistical significance was determined using Mann-Whitney test; ns (not significant), *(P < 0.05), **(P < 0.01).
