# Supplementary figures and images for: Vaccine induced memory CD8+ T cells efficiently prevent viral transmission from the respiratory tract
Source: Front Immunol. 2023 Dec 18;14:1322536. doi: 10.3389/fimmu.2023.1322536 (PMC10757911; doi:10.3389/fimmu.2023.1322536)

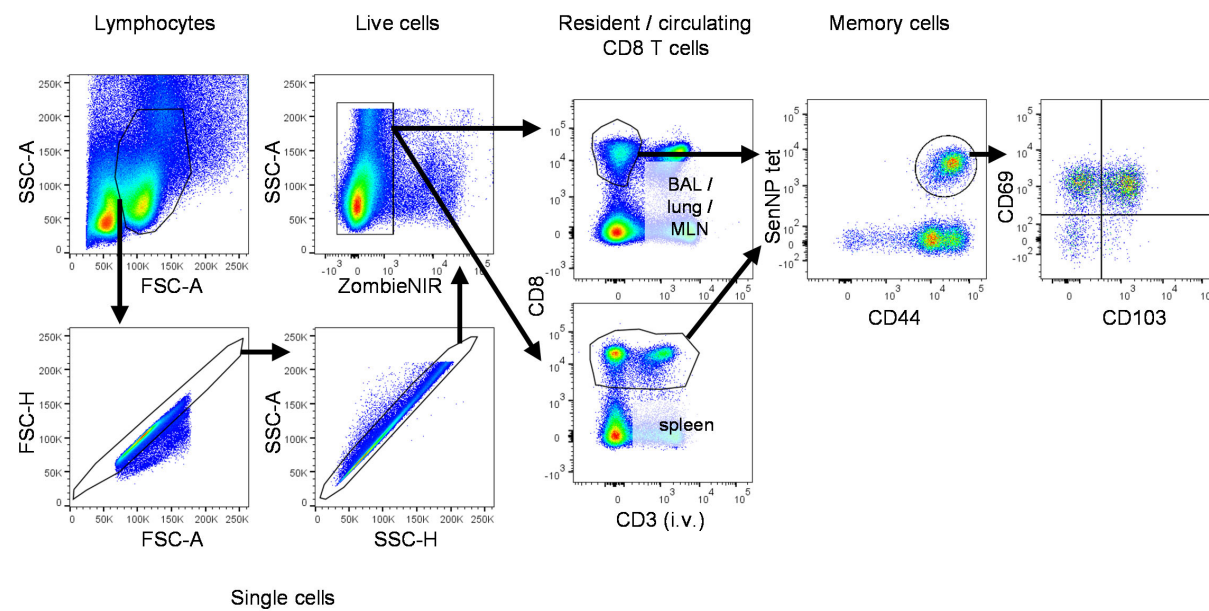

Supplementary fig 1

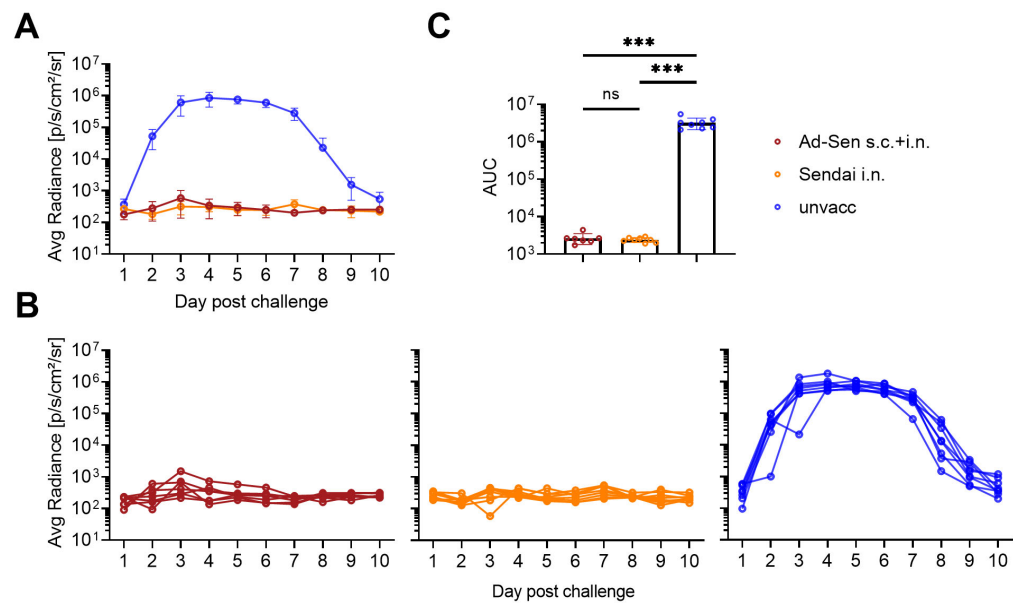

Supplementary fig 2

Supplement: Supplementary Figure 1 — Gating strategy. Lymphocytes were first gated on a forward area and side area plot. After gating single cells on a forward area and height plot and a side area and height plot, live cells were identified with ZombieNIR. Intravascular staining was used to discriminate circulating (i.v. CD3+) and resident (i.v. CD3-) cells in the lungs, BAL and MLN. SenNP+CD44+CD8+ cells were defined as antigen-specific (tetramer+) memory T cells and were then gated into a new plot with CD69 and CD103. [file Image_1.pdf]
